# Development of Quantitative Methylation-Specific Droplet Digital PCR (ddMSP) for Assessment of Natural Tregs

**DOI:** 10.3389/fgene.2020.00300

**Published:** 2020-04-07

**Authors:** Mohamed I. Husseiny, Ahmed Fahmy, Weiting Du, Angel Gu, Pablo Garcia, Kevin Ferreri, Fouad Kandeel

**Affiliations:** ^1^Department of Translational Research & Cellular Therapeutics, Diabetes & Metabolism Research Institute, Beckman Research Institute of City of Hope, Duarte, CA, United States; ^2^Faculty of Pharmacy, Zagazig University, Zagazig, Egypt; ^3^East Lancashire Hospitals NHS Trust, Blackburn, United Kingdom

**Keywords:** natural T regulatory cells, tissue-specific methylation, droplet digital PCR, methylation-specific PCR/quantitative methylation-specific PCR, demethylation of TSDR FOXP3

## Abstract

Regulatory T cells (Tregs) suppress immune responses *in vivo* in an antigen-specific manner. Of clinical relevance, Tregs can be isolated and expanded *in vitro* while maintaining immunoregulatory function. Tregs are classified as CD4^+^CD25^high^CD127^low^ FOXP3^+^ cells. Demethylation of the Treg-specific demethylation region (TSDR) of FOXP3 is found in natural Tregs (nTregs). We report a method for the characterization of the differential methylation pattern of the FOXP3 TSDR in patient-derived and expanded nTregs. Human TSDR sequences from nTregs (unmethylated sequence) and pancreatic (methylated sequence) cells were amplified and cloned into plasmids. A droplet digital TaqMan probe-based qPCR (ddPCR) assay using methylation-specific primers and probes was employed to quantify unmethylated and methylated sequences. The methylation-specific droplet digital PCR (ddMSP) assay was specific and selective for unmethylated DNA in mixtures with methylated DNA in the range of 5000 copies/μL to less than 1 copy/μL (*R*^2^ = 0.99) even in the presence of non-selective gDNAs. CD4^+^CD25^high^CD127^low^FOXP3^+^ human nTregs, in the presence of Dynabeads or activators, were expanded for 21 days. There was a decrease in the unmethylated ratio of Tregs after expansion with essentially the same ratio at days 10, 14, and 17. However, the activator expanded group showed a significant decrease in unmethylated targets at day 21. The suppression activity of activator-expanded nTregs at day 21 was decreased compared to cells expanded with Dynabeads. These data suggest that the ddMSP can quantitatively monitor nTreg expansion *in vitro*. These data also indicate that the assay is sensitive and specific at differentiating nTregs from other cells and may be useful for rapid screening of nTregs in clinical protocols.

## Introduction

Regulatory T cells (Tregs), previously known as suppressor T cells, are defined as cells that suppress the immune response (Bettelli et al., 2006). They play a critical role in maintaining immune tolerance (Chatenoud et al., 2001) and are essential to transplantation tolerance. Tregs prevent auto-activation of T cells that have escaped negative selection in the thymus. Most autoimmune diseases are associated with reduced numbers or decreased activity of Tregs (Kriegel et al., 2004; Viglietta et al., 2004; Offner and Vandenbark, 2005; Takahashi et al., 2006). Initially, Tregs were characterized as CD4^+^ T cells that express high level of CD25. However, it was noted that CD25 was induced in conventional T cells following stimulation limiting its use for characterization of Tregs (Sakaguchi, 2005).

Transcription factor Forkhead Box P3 (FOXP3) is essential for the normal development and function of natural CD4^+^CD25^high^CD127^low^FOXP3^+^Tregs (nTregs) (Walker et al., 2003; Yagi et al., 2004; Fontenot et al., 2005; Marie et al., 2005; Roncador et al., 2005). Human FOXP3 is the most widely used molecular marker for nTreg identification and characterization. T cells transiently upregulate FOXP3 following antigen stimulation but its function in this situation remain unclear (Wang et al., 2007). Stable expression of FOXP3 is found in rodent and human nTregs and DNA hypomethylation of the FOXP3 gene is required for its regulation (Baron et al., 2007; Floess et al., 2007). Three regions in the FOXP3 locus are epigenetically modified and involved in regulating FOXP3 expression namely the promoter, TGFβ sensor, and Treg-specific demethylated region (TSDR). The FOXP3 TSDR is a conserved CpG-rich region which is demethylated in nTregs but methylated in other cell types (Baron et al., 2007; Floess et al., 2007). Indeed, an epigenetic modification in the FOXP3 TSDR has been promulgated as a means of identifying Tregs (Baron et al., 2007; Begin et al., 2015; Rossetti et al., 2015).

Droplet digital PCR (ddPCR) enables TaqMan hydrolysis probe-based assays for the absolute quantification of nucleic acids (Ottesen et al., 2006; Warren et al., 2006; Fan and Quake, 2007). Recently, a ddPCR system compatible with both TaqMan-probe and DNA-binding dye detection chemistries was developed (McDermott et al., 2013). Importantly, ddPCR does not require generation of a standard curve or endogenous controls because it directly counts the number of target molecules (Pretto et al., 2015). Furthermore, ddPCR has been used for detection and quantification of circulating levels of DNA in the plasma of cancer patients (van Ginkel et al., 2017).

Clinical programs employing human Tregs could benefit from a quantitative method for *ex vivo* assessment of Treg expansion. We established a quantitative methylation-specific ddPCR (ddMSP) assay for assessment of TSDR methylation status in *ex vivo* expanded nTregs as a surrogate for nTreg stability. nTregs were expanded by several means and the degree of expansion characterized as the percentage of demethylation of TSDR FOXP3. We also applied this approach to evaluate the suppressive activity of the expanded nTregs.

## Materials and Methods

### Human Subjects

Human tissues including liver, pancreas and blood were obtained from the pathology laboratory at City of Hope under Institutional Review Board approval (IRB# 01083, 08078, 08079, and 05058). In other instances, written informed consent was obtained from donors for research use of the collected samples.

### Genomic DNA Isolation and Bisulfite Treatment

Genomic DNA (gDNA) was obtained from sorted nTregs and from *ex vivo* expanded nTregs using the DNeasy blood and tissue kit (QIAGEN, Valencia, CA, United States). The gDNA samples were bisulfite treated using the EZ-DNA methylation-gold kit (Zymo Research, Orange, CA, United States) according to the manufacturer’s recommendation.

### Standard Plasmids

Primers Amp5 forward and Amp5 reverse (Table 1) were used to amplify a 336 bp fragment of FOXP3 TSDR using human bisulfite-treated gDNA isolated from sorted nTregs or pancreatic tissue. The bisulfite-treated human gDNA isolated from sorted nTregs was a source of unmethylated FOXP3 TSDR and gDNA from pancreatic tissue for methylated FOXP3 TSDR. The PCR products were cloned into the pCR2.1-TOPO plasmid vector using the TOPO-TA cloning kit (Invitrogen, Carlsbad, CA, United States). The cloned sequences were confirmed using M13F and M13R primers by the DNA Sequencing/Solexa Core Facility at Beckman Research Institute of City of Hope. Plasmids were purified with the Qiagen Plasmid Midi Kit and diluted to obtain final concentrations of 10^6^, 10^5^, 10^4^, 10^3^, 10^2^, 10, and 1 copy/reaction as a standard for qPCR reactions for methylated and unmethylated FOXP3.

**TABLE 1:** Oligonucleotides used in this study.

	Designation	Sequence
**Primers for cloning the *Amp5* fragment**		
1	Amp5-forward	5′-TGTTTGGGGGTAGAGGATTT-3′
2	Amp5-reverse	5′-TATCACCCCACCTAAACCAA-3′
**Primers and probes for qMSP and qBSP**		
P17	UMTreg-For	5′-GTATTTGGGTTTTGTTGTTATAGTTTTT-3′
P18	UMTreg-Rev	5′-CTACAAAACAAAACAACCAATTCTCA-3′
P19	MTreg-For	5′-GTATTTGGGTTTTGTTGTTATAGTTTTC-3′
P20	MTreg-Rev	5′-TACAAAACAAAACAACCAATTCTCG-3′
P21	UMTreg-Probe with FAM	5′-GTGGTTGGATGTGTTGG-3′
P22	MTreg-Probe with VIC	5′-GCGGTCGGATGCGTCGG-3′
P24	MTreg-Probe with FAM	5′-CGACGCATCCGACCGCCA-3′
P25	UMTreg-Probe with VIC	5′-ACCCAACACATCCAACCACCA-3′
P26	BSTSDR-Treg-For	5′-GTTTGTATTTGGGTTTTGTTGTTATAG-3′
P27	BSTSDR_Treg-Rev	5′-CTACTACAAAACAAAACAACCAATTC-3′
P31	BSTSDR-Probe with FAM	5′-ATCTACCCTCTTCTCTTCCTC-3′

### Cell Sorting and Culture

Peripheral blood mononuclear cells (PBMCs) were isolated by Ficoll-Paque gradient centrifugation (GE Healthcare, Piscataway, NJ, United States) from the buffy coat. CD3^+^ T cells were then magnetically labeled and isolated by autoMACS^®^ Pro Separator (Miltenyi Biotec). CD3^+^ T cells were further stained with antibodies against CD4, CD25, and CD127 (BD Biosciences, San Jose, CA, United States) and CD4^+^CD25^+^CD127^–^ cells were sorted by a FACSAria III cell sorter (Du et al., 2013). Sorted Tregs were cultured in X-VIVO 20 (Lonza, Walkersville, MD, United States) media containing 10% human heat-inactivated AB serum (Valley Biomedical, Winchester, VA, United States) plus anti-CD3/CD28 Dynabeads (Invitrogen; Carlsbad, CA, United States) at a 1:1 cell-to-bead ratio or soluble CD3/CD28/CD2 T Cell Activator (Stem Cell Technology) at a ratio of 25 uL/1 million cells according to the manufacturer’s recommendation. At day 2, recombinant human IL-2 was added (500 units/mL, Peprotech, Rocky Hill, NJ, United States). Fresh media and IL-2 were added every 2–3 days. On day 7 and day 14, cells were re-stimulated with anti-CD3/CD28 beads or CD3/CD28/CD2 T Cell Activator. On day 21, the cultured cells were stained for CD4, CD25, and Foxp3.

### Suppression Assays

The suppressive function of expanded Tregs was determined using PBMCs labeled with carboxyfluorescein diacetate succinimidyl ester (CFSE, Invitrogen/Molecular Probes, Eugene, OR, United States) as targets. CFSE-labeled autologous PBMCs (1 × 10^5^) were incubated with various ratios of Tregs (1:1, 1:0.5, 1: 0.25, 1:0.125, and 1:0) in the presence of anti-CD3/CD28 Dynabeads. On day 3, cells were harvested and stained with anti-CD8 to assess cell proliferation. To further evaluate the function of expanded Tregs, the percentage of suppression was calculated using the following formula: (% CFSE-labeled CD8^+^ T cells–% Treg-co-cultured CFSE-labeled CD8^+^ T cells)/(% CFSE-labeled CD8^+^ T cells) × 100 (Lyons and Parish, 1994; Du et al., 2013; Ward et al., 2014).

### Flow Cytometry

The phenotype of expanded Tregs was determined by staining with the indicated antibodies and analyzed using a FACSCanto II system (BD). Antibodies to human CD4-PerCp (Clone SK3), CD25-APC (clone 2A3), and CD127-PE (Clone HIL-7R-M21) were from BD, and Foxp3-Alexa Fluor^®^ 488 (clone 206D) from BioLegend. Foxp3 staining was performed using the Foxp3 Fix/Perm Buffer kit (BioLegend). FACS data were analyzed using Flowjo software (Treestar, Ashland, OR, United States).

### Droplet Digital Methylation-Specific PCR Assay

Approximately 10–50 ng of bisulfite-treated DNA per sample was used in the ddPCR reaction. VIC fluorescent-tagged TaqMan assay probe was used to quantify the copy number of the unmethylated FOXP3TSDR of Tregs. FAM fluorescent-tagged TaqMan assay probe was used to quantify the methylated FOXP3TSDR of Tregs. The ddPCR system was operated according to the manufacturer’s instruction (Pretto et al., 2015; Fujiki et al., 2016). Briefly, the PCR reaction solution was dispensed into a single well on a 96-well plate containing ddPCR Supermix for Probes (no dUTP) (Bio-Rad), 900 nmol/L primers, and 250 nmol/L probe in a final volume of 25 μL. Primers and probes sequences (Life Technologies) are listed (Table 1). The generation of droplets was performed using 20 μL of the assay mix and 70 μL of droplet generation oil pipetted into a QX200 DG cartridge (Bio-Rad), then loaded into a QX200 Droplet Generator (Bio-Rad). Generated droplets were carefully transferred into a 96-well PCR plate and the plate was heat-sealed with foil using a PX1 Plate Sealer (Bio-Rad). The PCR reactions were performed using the C100 Touch Thermal Cycler (Bio-Rad) with the following PCR conditions: 10 min enzyme activation at 95°C followed by 40 cycles of denaturation at 94°C for 30 s and annealing-extension at 55°C for 1 min. An enzymatic-deactivation step was included at the end at 98°C for 10 min and plates were stored at 10°C until droplets were counted using a Bio-Rad QX200 Droplet Reader (Bio-Rad). After the PCR amplification was completed, a droplet reader counted the number of droplets that were positive or negative for FAM and VIC fluorophore. ddPCR was run in triplicates. No-template controls were included in each run to control for contamination during reaction. Analysis of data was performed with QuantaSoft analysis software (Bio-Rad) that accompanied the QX200 Droplet Reader.

### Statistical Analysis

Statistical significance between samples was tested with a two-tailed Student’s *t*-test for paired values or two-way analysis of variance (ANOVA) using GraphPad Prism 7 software. Statistical significance was stratified as a *p*-value of <0.05, <0.01, and <0.001.

## Results

### Overview of Development of Methylation-Specific and Probe-Dependent Droplet Digital PCR

**FIGURE 1:**
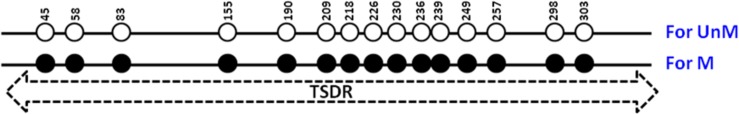
Human TSDR of FOXP3. Schematic illustration of the amp-5 region of human TSDR of FOXP3 is showing the position of the 15 CpG sites. Solid circles represent methylated CpGs and open circles represent unmethylated CpGs. UnM, unmethylated; M, methylated.

We reported a quantitative SYBR Green-based PCR assay to discriminate between methylated and unmethylated CpG containing DNA (Husseiny et al., 2012, 2014). In this study, we tested the hypothesis that this technology could be extended to develop a quantitative PCR assay to quantify the unmethylated TSDR of FOXP3 of nTregs. We chose the Amp 5 region of the TSDR of FOXP3 that has 15 CpGs (Figure 1) that are differentially unmethylated and specific for the TSDR of FOXP3 of nTregs but that are methylated in other cell types (Baron et al., 2007). For this purpose we cloned bisulfite-treated gDNA of the Amp 5 sequence from human nTregs, corresponding to unmethylated target, and pancreatic cells, corresponding to methylated TSDR of FOXP3 (Figure 1). The quantitative methylation-specific PCR (qMSP) consisted of methylation-dependent amplification primers and hybridization probes for both bisulfite-converted methylated and unmethylated DNA of the TSDR of FOXP3 (Figure 1). We combined the methylation-specific probes-dependent PCR system with the ddPCR system (Pretto et al., 2015) to develop a sensitive and accurate PCR reaction.

### Standard Curves and Specificity of the Reaction

**FIGURE 2:**
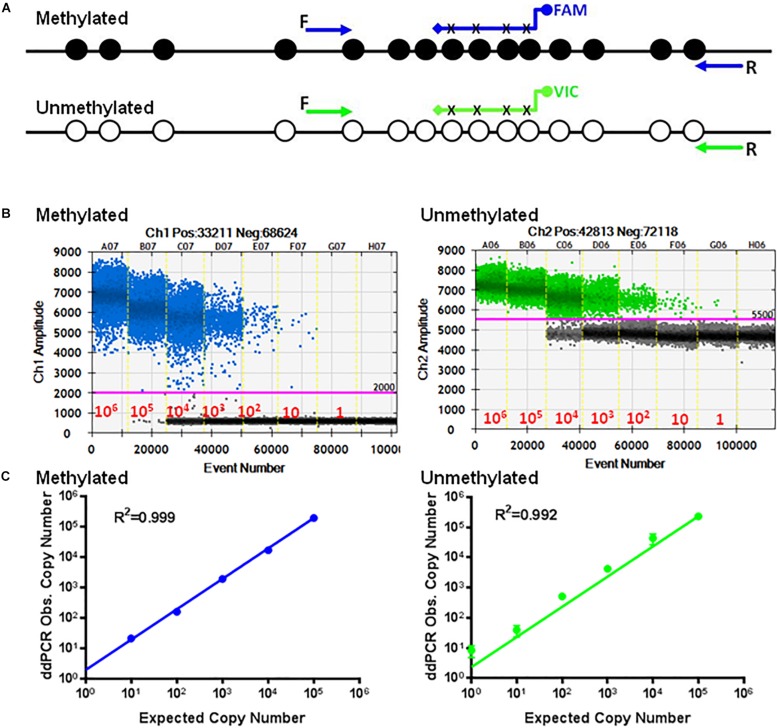
Standard curves of methylated and unmethylated FOXP3 TSDR. **(A)** Schematic illustration of the TSDR of FOXP3 showing the specific primer set for methylated CpGs with specific probes conjugated with FAM and the specific primer set for unmethylated CpGs with specific probes conjugated with VIC that interrogate six CpGs of TSDR. **(B)** ddPCR using serially diluted plasmids (10^6^–1 copy/reaction). **(C)** Standard curves with linear regression analysis for methylated and unmethylated targets. The data shown are the average with standard deviation (SD) of three repeats.

Figure 2A shows 15 CpGs located on the human TSDR of the FOXP3 sequence. Specific primers and probes were designed to match the unmethylated CpG sequences at positions 190 (P17), 303 (P18) at the 3 prime end and probes at 226, 230, 236, and 239 (P25) (Table 1). This primer set, along with the probes, can only detect the unmethylated TSDR of FOXP3. Conversely, primers P19 and P20 and probe P24 (Table 1) can only detect the methylated TSDR of FOXP3.

Employing ddPCR, the primer sets were evaluated using serial dilutions of the cloned methylated and unmethylated TSDR of FOXP3 DNA as templates. As shown, each UnMSP and MSP reaction exhibited dose-dependent amplification ranging from 10^6^ copies to 1 copy of the unmethylated and methylated sequences (Figure 2B). Quantitative analysis of the standard curves showed that the UnMSP (*R*^2^ = 0.992) and MSP (*R*^2^ = 0.999) assays were linear over a 10^6^-fold range of template concentrations (Figure 2C). In comparison, the quantitative analysis of the standard curves for conventional UnMSP (*R*^2^ = 0.898, slope = −2.992) and MSP (*R*^2^ = 0.978, slope = −3.005) assays were less linear especially for the UnMSP assay (Supplementary Figure 1).

**FIGURE 3:**
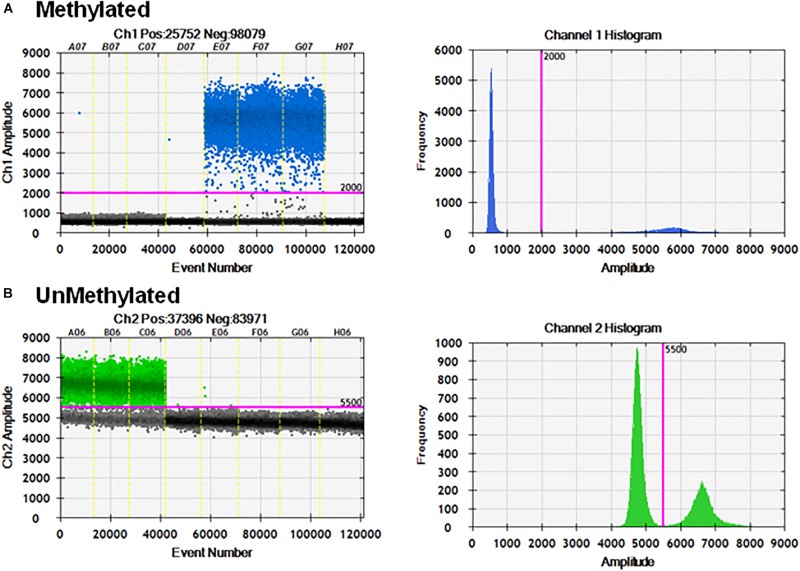
Specificity of ddMSP assay. ddPCR in the presence of mixtures of methylated and unmethylated plasmids. **(A)** Methylation probe and primers can only detect methylated CpGs and cannot detect unmethylated CpGs. **(B)** Unmethylation probe and primers can only detect unmethylated CpGs and cannot detect methylated CpGs.

The primer sets and probes were tested for specificity and cross-reactivity with the opposite templates. The primer set and probes specific for unmethylated template can only detect unmethylated CpG-containing sequences (Figure 3).

### Standard Curves for the Multiplex ddPCR

**FIGURE 4:**
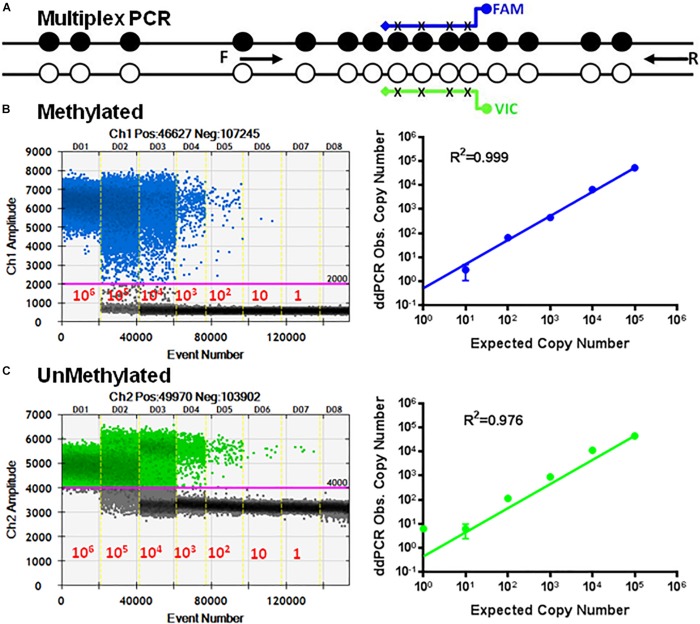
Multiplex ddMSP assay. **(A)** Schematic illustration of the TSDR of FOXP3 showing two general TSDR primers with probes for each of the methylated and unmethylated CpGs that interrogates four CpGs of TSDR. Standard curves of methylated **(B)** and unmethylated **(C)** FOXP3 TSDR in the same reaction. ddPCR using serially diluted mixed plasmids (10^6^–1 copy/reaction). Standard curves with linear regression analysis for methylated and unmethylated targets. The data shown are the average with standard deviation (SD) of three repeats.

Next, we developed multiplex ddPCR to detect both unmethylated and methylated CpGs in the same reaction (Figure 4). To do this we replaced primers P17, P18, P19, and P20 with the primers P26 and P27 (Table 1) in combination with probes P24 and P25. The sequences for P26 and P27 are bisulfite-specific and not methylation-specific (Figure 4B). This mean that P26/P27 with the probe P24 only detects methylated CpG sequences, while P26/P27 with the probe P25 only detects unmethylated CpG sequences (Figure 4C).

The primer set P26/P27 in combination with probes P24/P25 was evaluated using serial dilutions of equal mixtures of the cloned methylated and unmethylated TSDR of FOXP3 DNA using ddPCR. Figure 4 shows dose-dependent amplification ranging from 10^6^ to 1 copy of unmethylated and methylated sequences. The standard curves derived from these reactions showed linearity for UnMSP (*R*^2^ = 0.976) and MSP (*R*^2^ = 0.999).

### Specificity of the Probe on Standard Curves in the Multiplex Assay

**FIGURE 5:**
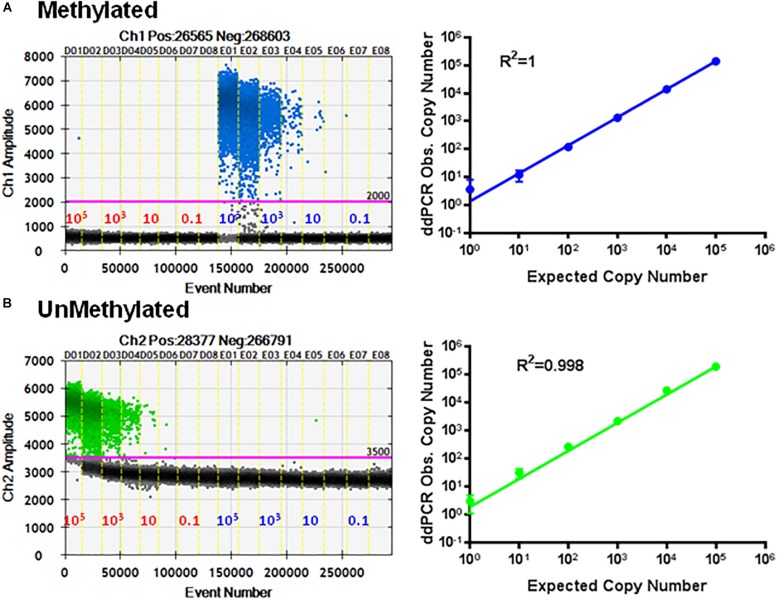
Specificity of ddMSP assay. Standard curves of methylated **(A)** and unmethylated **(B)** FOXP3 TSDR in the same reaction. ddPCR using serially diluted mixed plasmids (10^5^–0.1 copy/reaction) and two TSDR-specific primers with probes for methylated and unmethylated CpGs. Standard curves with linear regression analysis of methylated and unmethylated targets. The data shown are the average with standard deviation (SD) of three repeats.

To check the specificity of each probe, the primer set P26/P27 and probes P24/P25 were evaluated using serial dilutions of cloned methylated and unmethylated TSDR of FOXP3 DNA separately. Figure 5A shows linear amplification in a standard curve for methylated PCR (*R*^2^ = 1) without detection of unmethylated CpG sequences. Also, amplified unmethylated CpG sequences displayed a linear relationship (*R*^2^ = 0.998) without detection of methylated CpGs (Figure 5B).

### Effect of gDNA Background on the Specificity and Sensitivity of the Multiplex Assay

**FIGURE 6:**
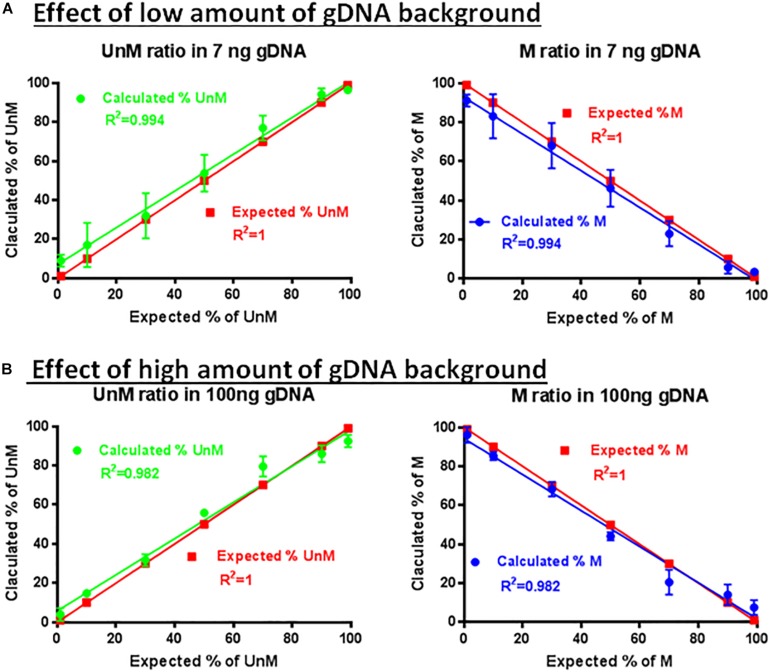
Effect of non-specific gDNA on the specificity and sensitivity of ddMSP. Combinations of different ratios of unmethylated/methylated plasmids (1/99, 10/90, 30/70, 50/70, 70/30, 90/10, and 99/1) in the presence of low **(A)** or high amounts **(B)** of non-specific gDNA as background. Mixture analyzed by ddPCR using primers specific to TSDR and probes for methylated and unmethylated CpGs. Linear regression analysis for methylated and unmethylated targets showed no effect of non-specific background. The data shown are the average with standard deviation (SD) of three repeats.

The specificity and sensitivity of the developed assay was first confirmed by using methylated and demethylated TSDR of FOXP3-cloned plasmid sequences (Figure 6). The ability of the primer sets and probes to detect the methylated or unmethylated DNA in the presence of large amounts of non-specific gDNA was assessed. The primer set P26/P27 and probes P24/P25 were used for amplification using different percentages of the methylated (1, 10, 30, 50, 70, 90, and 99%) and unmethylated (99, 90, 70, 50, 30, 10, and 1%) TSDR of FOXP3 DNA in the presence of low (Figure 6A) or high (Figure 6B) amounts of non-specific gDNA. After performing ddPCR, the copy numbers of unmethylated and methylated DNA were calculated followed by the percentage of each to the total DNA. Comparing Figures 6A,B, the primer set (P25/P27) with probe P25 can detect as little as 1% of unmethylated, or with the probe P24, methylated templates in the presence of non-specific gDNA background. Furthermore, as shown in Figure 6, the primer set with the probes was largely unaffected by the presence of the non-specific gDNA and was able to detect as little as 10 or fewer copies of unmethylated or methylated templates, but did not produce a detectable signal from the non-specific gDNA background.

In some cases, rather than using a primer set with a probe that measured the copy numbers of methylated gDNA, we used a probe that measured the whole gDNA, such as probe P31 (Table 1). Under these conditions, the multiplex assay measured the percentage of the unmethylated TSDR of FOXP3 related to the total gDNA.

### The ddMSP Assay for Assessment of Expanded nTregs

The translational relevance of the ddMSP assay was assessed using CD4^+^CD25^high^CD127^low^ T cells isolated from the PBMCs of healthy donors. Flow cytometry analysis revealed FOXP3 expression in >95% of CD25^high^ Tregs (Supplementary Figure 2A). Interestingly, Treg FOXP3 was found to be primarily methylated with a mere 5% demethylated and more than 90% demethylated after sorting (Supplementary Figure 2B). In these studies, the primer set P26/P27 and probes P25 and P31 (Table 1) were used to amplify the percentage of the unmethylated TSDR of FOXP3 of Tregs to the total DNA.

**FIGURE 7:**
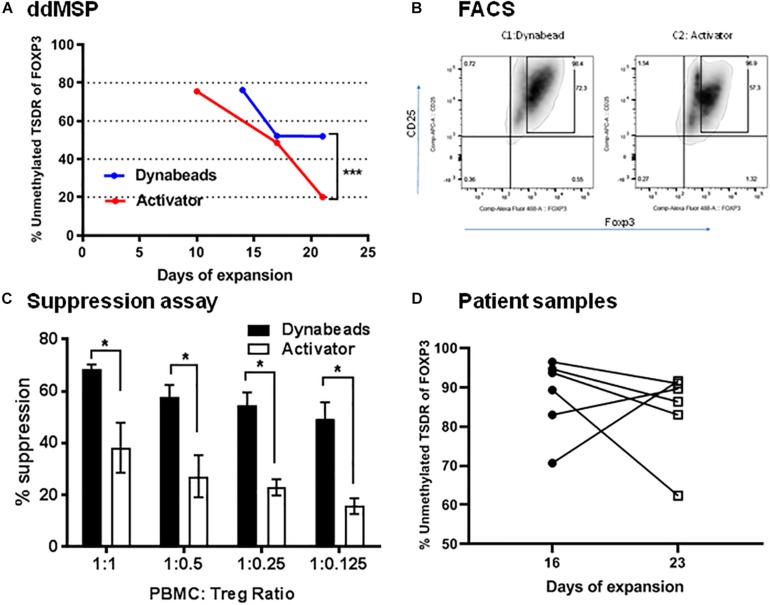
Validation of ddMSP assay. Human PBMCs were enriched with anti-CD3 and then sorted into CD4^+^CD25^high^CD127^low^Tregs by FACS. Sorted nTregs expanded by Dynabeads or activator in the presence of human IL-2. **(A)** Percentage of unmethylated TSDR of FOXP3 for expanded nTregs at 10, 14, 17 and 21 days of expansion. Statistical analysis using Wilcoxon matched-pairs rank test shows the significance between Dynabeads and activator (***p < 0.001). The data shown are the average with SD of three repeats. **(B)** Representative FACS plots gated on CD4 T cells indicate the percentage of CD4^+^CD25^+^Foxp3^+^ T cells at day 21. **(C)** Suppression assay. CFSE-labeled PBMCs were co-cultured with Dynabeads or activator-expanded nTreg from the same donor at various ratios. The cells were stimulated with anti-CD3/anti-CD28 Dynabeads for 3 days, and the proliferation of CFSE-labeled CD8^+^ T cells was analyzed by FACS. Statistical analysis using two-way ANOVA shows the significance between Dynabeads and activator (*p < 0.05). The data shown are the average with SD of three repeats. **(D)** Quantification of the percentage of unmethylated TSDR of FOXP3 in expanded Treg cells after 16 and 23 days of expansion using the ddMSP assay. The data shown are the average with standard deviation (SD) obtained from six different donors and three repeats.

Using Dynabeads or activator, the effects on *ex vivo* Tregs expansion in the presence of human IL2 and sirolimus were also tested. Dynabead-expanded Tregs were collected at days 14, 17 and 21 whereas activator-expanded Tregs were collected at days 10, 17, and 21. Tregs had lost demethylation of TSDR after expansion regardless of conditions (Figure 7A). Specifically, Dynabead-expanded Tregs displayed levels of the demethylated TSDR of 80% at day 14, 51% at day 17 and 50% at day 21. Activator-expanded Tregs levels of demethylated TSDR were 80% at day 10, 50% at day 17 and 20% at day 21. In a comparative study, flow cytometry analysis revealed that FOXP3 was expressed in 72% of CD25^high^ Tregs expanded by Dynabeads and 57% in CD25^high^ Treg expanded by activator (Figure 7B).

Changes in methylation/demethylation at day 21 altered Treg functionality. Indeed, Tregs with demethylated TSDR of FOXP3 were more suppressive of effector T cells than Tregs expanded by activator (Supplementary Figure 3) that lost demethylated target (Figure 7C). Furthermore ddMSP assays were performed using sorted CD4^+^CD25^high^CD127^low^ T cells isolated from the PBMCs of an additional group of healthy donors (*n* = 6). These sorted Tregs were expanded *ex vivo* and characterized by quantifying the percentage of demethylation after 16 and 23 days of expansion (Figure 7D). The results showed that, after 23 days of expansion, demethylation of three out of six Treg samples was not affected. In contrast, one expanded Treg cell sample was noted to have demethylation decreased from 90 to 60%. Also, in two cases expanded Treg cells displayed increased demethylation from 70 or 80% to above 90% (Figure 7D).

Furthermore on assessing the reproducibility of the ddMSP assay, we found it to be highly reproducible as the coefficient of variation of the assay performed for the six samples was approximately 1% at day 16 of expansion and roughly 1 to 2% at day 23 of expansion (Table 2).

**TABLE 2:** Reproducibility of the qddMSP assay.

Donor	Day 16 of expansion						Day 23 of expansion					
	% Demethylation repeats			Average	±SD	%CV	% Demethylation repeats			Average	±SD	% CV
1	82	83	84	83.0	1.00	**1.20**	89	91	89	89.67	1.15	**1.29**
2	96	97	95	96.0	1.00	**1.04**	91	92	93	92.00	1.00	**1.09**
3	94	93	94	93.7	0.63	**0.67**	82	83	84	83.00	1.00	**1.20**
4	89	89	90	89.3	0.58	**0.65**	66	64	63	64.33	1.53	**2.37**
5	71	70	72	71.0	1.00	**1.41**	91	92	92	91.67	0.58	**0.63**
6	94	95	95	94.7	0.58	**0.58**	88	86	85	86.33	1.53	**1.77**

*Replicate assays were done on two separate time point, and each assay represents the mean data for at least three repeats. SD is the standard deviation, and % CV is the percent coefficient of variation [(SD/average)×100].*

## Discussion

Regulatory T cell therapy is a promising approach for transplant rejection and severe autoimmunity. However, the therapeutic application of Tregs requires *ex vivo* expansion to provide sufficient cell mass. Prior to widespread application, technical issues need to be resolved including the stability of cultured Tregs, and loss of functionality after expansion. Also, current methods of quantifying Tregs by assessing FOXP3 and CD25 expression are inadequate (Roncador et al., 2005; Loddenkemper et al., 2006).

Demethylation status of the FOXP3 TSDR, in contrast to demethylation of the inducible promoter demethylated region (IPDR) of FOXP3 (Begin et al., 2015), is thought to be specific, stable and highly suitable for the quantitative measurement of nTregs (Baron et al., 2007; Floess et al., 2007; Tatura et al., 2015). Several studies characterized Tregs by quantification of demethylation of the TSDR in the FOXP3 using methylated-based real time (RT-PCR) assay (Wieczorek et al., 2009; Sehouli et al., 2011; Barzaghi et al., 2012; Rodriguez-Perea et al., 2015). Additionally, demethylation of TSDR of FOXP3 of Tregs was associated with the suppressive activity on the effector cells (Liu et al., 2010). Previously, we established a qMSP assay to differentiate between methylated and unmethylated insulin DNA released into the blood upon beta cell death (Husseiny et al., 2012; Husseiny et al., 2014). Extending this, a quantitative assay that combined methylation-specific and TaqMan probe-based assays and ddPCR was developed. The advantage of ddPCR is that it is technically simple and it provides an absolute quantification of the target DNA without the need for standard references (Wiencke et al., 2014; Butchbach, 2016).

Several other advantages of the ddMSP assay include increased specificity, sensitivity and reproducibility compared to traditional qPCR assay. Quantitative MSP is sensitive and specific for detection of circulating rare DNA (Kristensen et al., 2008). Our results indicate that the ddMSP assay is highly sensitive being able to detect from 10^6^ to 1 copy of unmethylated or methylated FOXP3 TSDR. This mean the limit of detection of the ddMSP assay is in the range of 5000 copies/μL to less than 1 copy/μL (*R*^2^ = 0.99). The assay is also very specific as the unmethylated probe can only detect unmethylated template while the methylated probe can only detect methylated template. Further, the multiplex assay can be completed in a minimum of time, is cost-effective, and less likely to introduce operator error. Recently, ddPCR was used to quantify the demethylated CpG promoter sites of the CD3Z gene that can be used to estimate the T cell numbers in human blood and tissue (Wiencke et al., 2014). Our newly developed ddMSP assay is predicted to be applicable for routine application and may accelerate standardization of Treg testing. One of the limitations of using the RT-PCR assay is that baseline levels of FOXP3 TSDR demethylation in Tregs vary between men and women because of X-chromosome inactivation (Wieczorek et al., 2009; Rossetti et al., 2015). The ddMSP assay can differentiate between the sources of nTregs by gender (Supplementary Figure 4). Further, FACS analysis detects all T cells, even those with transient FOXP3 expression. This is in contrast with the ddMSP assay that detects only Tregs with stable FOXP3 expression. Additionally, the ddPCR assay allows quantification of low expressing/abundant genes with excellent precision compared with qPCR (Taylor et al., 2017).

## Conclusion

In conclusion, a quantitative ddMSP assay in which ddPCR was combined with a probe-based assay is described and validated. The assay was found specific for quantitative analysis of nTregs. Furthermore, the methylation status of TSDR of FOXP3 was found to relate well to the *ex vivo* functionality of Tregs. Although we established a qMSP assay to quantify the DNA released into the circulation from dying beta cells, we believe that the ddMSP assay is more sensitive and specific and can be applied for human blood to quantify the changes in the Tregs in diabetic patients or patients with other diseases. In addition, this assay may be applied to monitor the Treg changes after *in vivo* expansion. Finally, the assay performed well using human samples and, given its simplicity and rapidity, may find use in the clinic.

## Data AvAilability Statement

The datasets generated for this study are available on request to the corresponding author.

## Ethics Statement

The studies involving human participants were reviewed and approved by Institutional Review Board approval of City of Hope. The patients/participants provided their written informed consent to participate in this study.

## Author Contributions

MH, AF, and KF conceived and designed the experiments. MH, AF, WD, AG, and PG performed the experiments. MH and FK analyzed the data. MH, KF, and FK wrote the manuscript.

## Conflict of Interest

The authors declare that the research was conducted in the absence of any commercial or financial relationships that could be construed as a potential conflict of interest.
