# Supplementary material for: Development of Quantitative Methylation-Specific Droplet Digital PCR (ddMSP) for Assessment of Natural Tregs
Source: Front Genet. 2020 Apr 7;11:300. doi: 10.3389/fgene.2020.00300 (PMC7154152; doi:10.3389/fgene.2020.00300)
Supplement: Supplementary file 1 [file Data_Sheet_1.pdf]

Development of Quantitative Methylation-Specific Droplet Digital PCR (ddMSP) for Assessment of Natural Tregs

Mohamed I. Husseiny [1, 2, *], Ahmed Fahmy [3], Weiting Du [1], Angel Gu [1], Pablo Garcia [1], Kevin Ferreri [1], and Fouad Kandeel [1]

[1] Department of Translational Research & Cellular Therapeutics, Diabetes & Metabolism Research Institute, Beckman Research Institute of City of Hope, Duarte, California 91010-3000, USA

[2] Faculty of Pharmacy, Zagazig University, Zagazig, Egypt

[3] East Lancashire hospitals NHS Trust, Blackburn, United Kingdom

***Corresponding author:**
Mohamed I. Husseiny
E mail: melsayed@coh.org

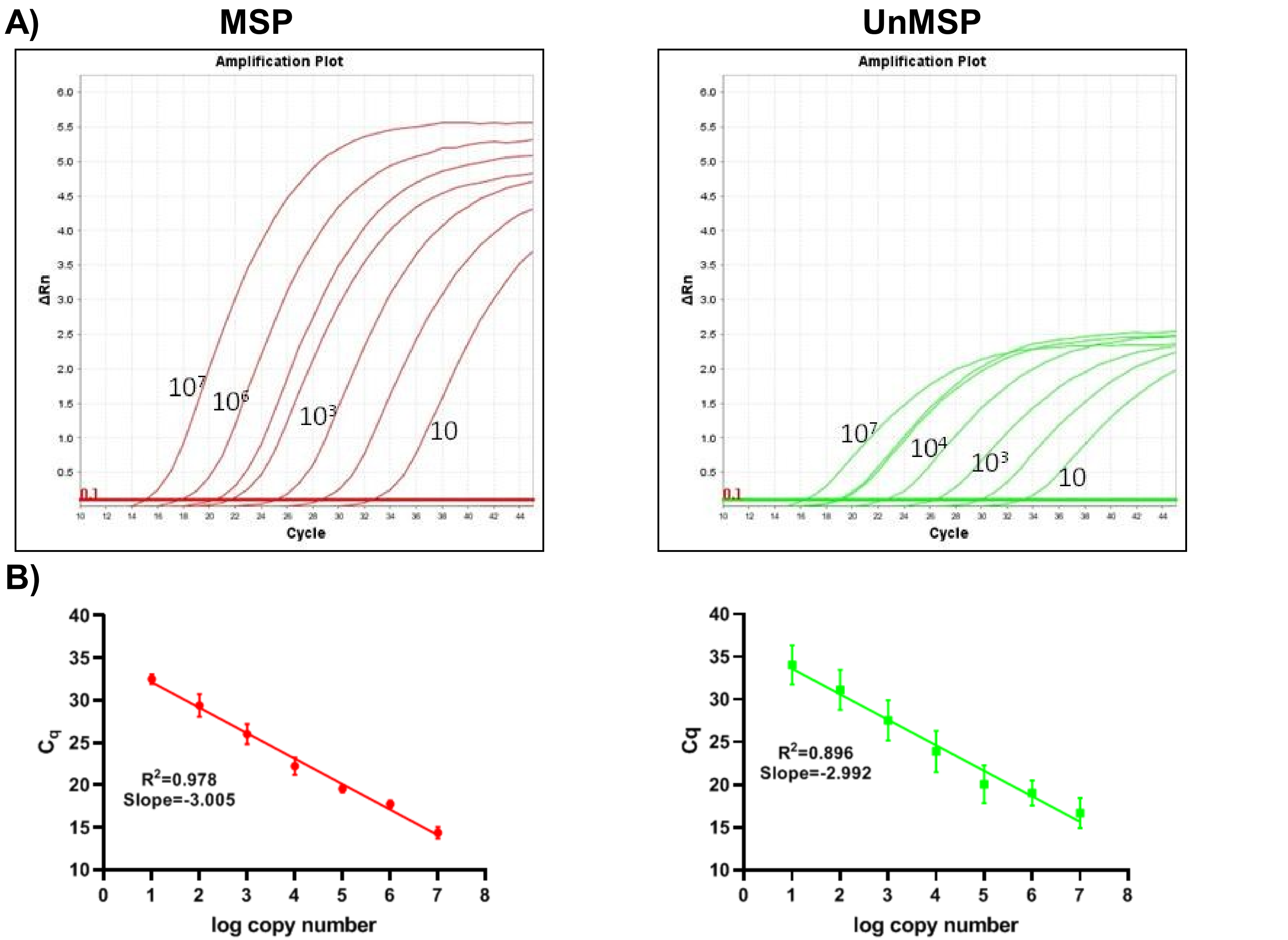

Supplementary Figure 1. Analytical performance of qMSP assay. **(A)** Amplification plots of a ten-fold serial dilution of methylated and unmethylated plasmids showing the fluorescence versus cycle using Primes (P19, P20) and Probe P22 for MSP and (P17, P18) with Probe 21 for UnMSP (Table 1). **(B)** Standard curves with linear regression analysis for methylated and unmethylated targets . The data showing Cq versus log copy number of serially diluted bisulfite-treated methylated or unmethylated plasmids. The data are the average with standard deviation (SD) of 4 repeats.

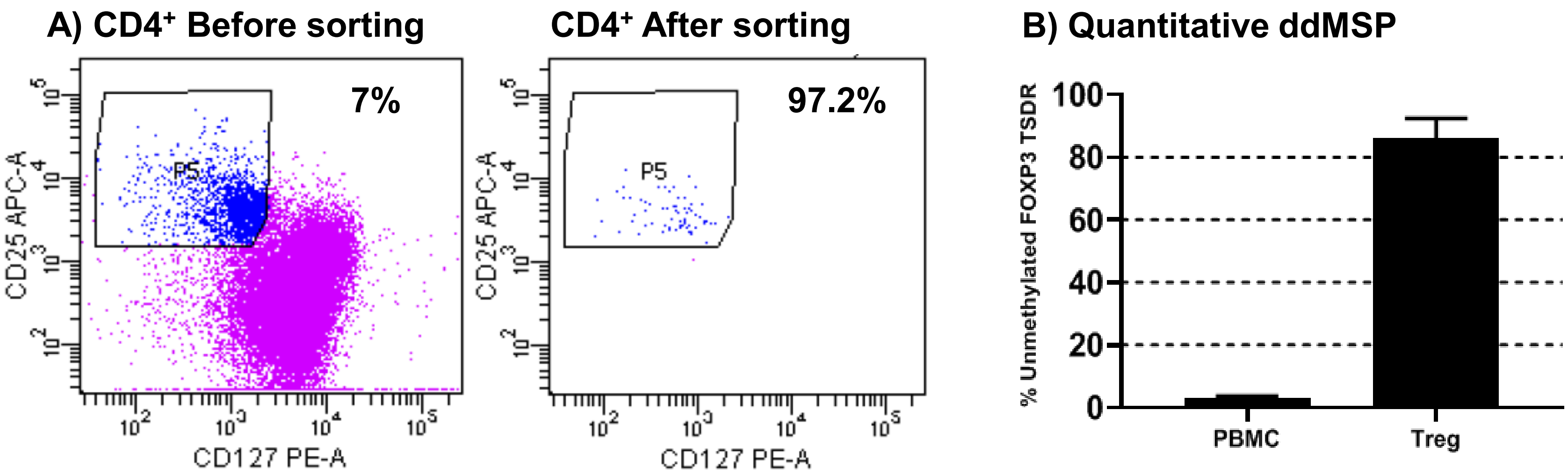

Supplementary Figure 2. Human PBMCs were enriched with anti-CD3 then sorted into $CD4^{+}CD25^{high}CD127^{low}$Tregs by FACS. **(A)** Flow cytometric plot gated on live $CD4^{+}$ T cells indicate the percentage of $CD25^{high}CD127^{low}$T cells in PBMC before and after sorting. **(B)** Quantification of the percentage of unmethylated TSDR of FOXP3 in PBMC and after sorting of Treg cells using ddMSP assay. The data shown are the average with standard deviation (SD) of 6 repeats from 2 different donors.

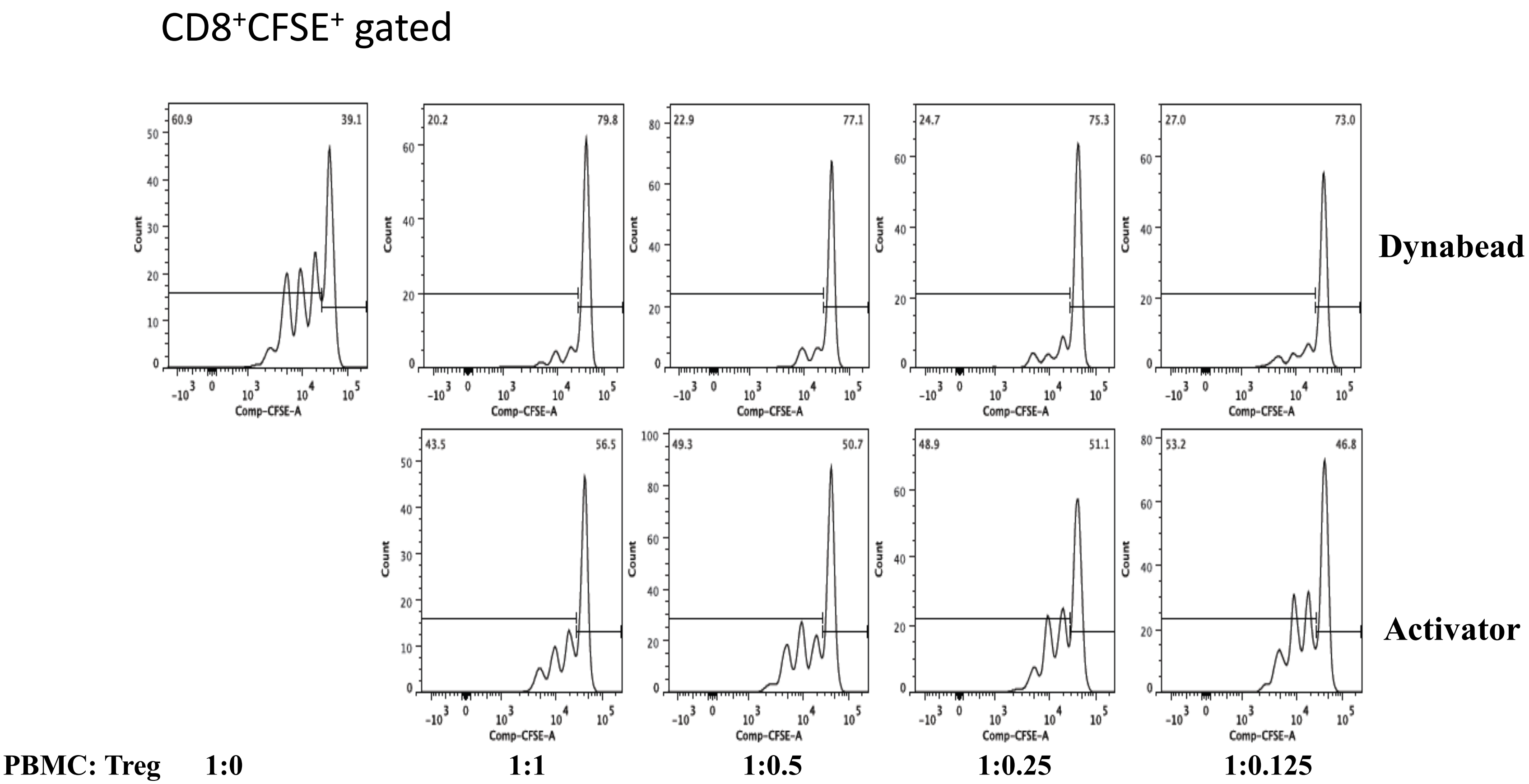

Supplementary Figure 3. **Suppression assay**. At day 21 of in vitro expansion of Tregs, CFSE-labeled PBMCs were co-cultured with Dynabeads or activator-expanded nTreg from the same donor at various ratios (PBMC/Treg=1:0, 1:1, 1:0.5, 1:0.25 or 1:0.125). The cells were stimulated with anti-CD3/anti-CD28 Dynabeads for 3 days, and the proliferation of CFSE-labeled $CD8^+$ T cells was analyzed by FACS after staining of CD8.

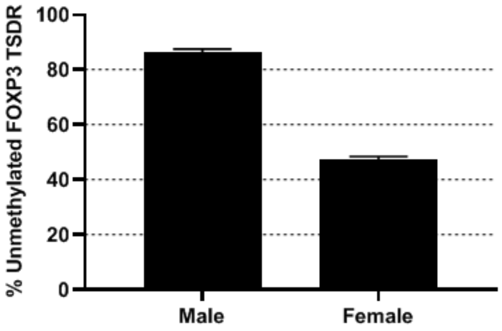

Supplementary Figure 4. Quantification of the percentage of unmethylated TSDR of FOXP3 in sorted Treg cells using ddMSP assay. The data shown are the average with standard deviation (SD) obtained from 4 males and 4 females.
